# Establishment and Clinical Application of the General Comfort Scale for Postoperative Lung Cancer Patients

**DOI:** 10.7759/cureus.49415

**Published:** 2023-11-26

**Authors:** Lisha Jiang, Mingming Wang, Guowei Che

**Affiliations:** 1 Day Surgery Center, West China Hospital of Sichuan University, Chengdu, CHN; 2 Department of Cardio-Thoracic Surgery, Chengdu Second People's Hospital, Chengdu, CHN; 3 Lung Cancer Center/Department of Thoracic Surgery, West China Hospital of Sichuan University, Chengdu, CHN

**Keywords:** general comfort questionnaire, lung cancer, enhanced recovery after surgery, delphi method, postoperative comfort scale for lung cancer patients

## Abstract

Background and purpose

The concept of enhanced recovery after surgery (ERAS) not only reflects rapid perioperative recovery but also focuses on the comfort experience of inpatients. This study intends to establish a clinically applicable general comfort questionnaire (GCQ) for patients with lung cancer after surgery and verify its clinical application effect.

Methods

The comfort index items for postoperative lung cancer were formed by combining previous research and literature, clinically applied comfort scales, and expert interviews. The Delphi method was used to conduct two rounds of expert consultations to determine the final index and establish a postoperative comfort scale for lung cancer patients. This scale was used to conduct a questionnaire survey on 200 patients to test the reliability and validity of the scale.

Results

The comfort scale contains 3 dimensions and 10 items and is easy to operate and evaluate in clinical applications. The Cronbach's α coefficient of the comfort scale is 0.801, and the scale content validity index (SCVI/ave) is 0.97. The common factor 1 and 2 characteristic roots of scale structural validity evaluation are 3.257 and 1.352 respectively, both greater than 1, with cumulative variance contribution rates of 32.57% and 13.52%. Pain and getting out of bed are the main factors influencing patient comfort.

Conclusion

The postoperative comfort scale for lung cancer patients has high clinical application reliability and validity. This study identified pain and mobility (early ambulation or getting out of bed) as the primary factors influencing the postoperative comfort of lung cancer patients.

## Introduction

The development of minimally invasive surgery has undoubtedly reduced the occurrence of trauma and complications at the technical operation level, leading to more rapid patient recovery. However, it is important to note that evaluating the quality of medical care and the "recovery" of patients should not solely focus on indicators such as the average length of hospital stay and the incidence of postoperative complications. It is equally crucial to consider the perioperative psychological well-being and emotional experiences of patients, which are often overlooked [1-3]. The concept of medical services and enhanced recovery after surgery (ERAS) is centered around providing "patient-centered" humanistic care. Therefore, it is crucial to prioritize the patient's comfort and experience during hospitalization in order to enhance patient satisfaction [1]. According to a survey, the average number of physical and mental symptoms after lung cancer surgery can reach as high as 8.3 [4]. Among these symptoms, pain and cough are the primary factors that directly impact the comfort of patients during their hospital stay [5]. The general comfort questionnaire (GCQ), developed by Kolcaba et al. based on the study of comfort theory [6], is a widely cited tool for measuring comfort. It has been translated and tested for reliability and validity by domestic scholars and is now widely used in the Chinese language [7-9]. The content of the comfort scale comprises four dimensions: physical, psychological, socio-cultural, and environmental, encompassing a total of 30 questions. Nonetheless, during its application to patients with postoperative lung cancer, it was observed that the GCQ scale contained an excessive number of items and lacked close alignment with the actual conditions experienced by postoperative patients. Consequently, this study performed optimizations on the GCQ scale, resulting in the development of a postoperative comfort scale tailored specifically for lung cancer patients. The validity and reliability of this scale were confirmed through its clinical application.

## Materials and methods

Objects and methods

Expert Selection

To ensure the validity and comprehensiveness of this research, experts from various fields were invited as consultation objects. The selection of these experts followed the principles of authority, representativeness, and voluntariness. Specifically, experts in the fields of thoracic surgery, nursing, rehabilitation medicine, mental health, and nutrition were invited to participate in this study. The criteria for selecting these experts were based on their expertise, experience, and willingness to contribute to the research. Expert selection criteria: (1) Have more than five years of working experience in a tertiary hospital; (2) Have a bachelor's degree or above, and have a senior professional title or above; (3) Be familiar with clinical work on lung cancer surgery patients and have rich theoretical foundation and practical experience; (4) Follow the principles of informed consent and voluntary participation and be able to complete the consultation questionnaire in a timely manner. Initially, 22 medical staff with senior professional titles from 14 medical centers were selected. They selected 10 comfort-related questionnaire items from three dimensions: self-care ability, postoperative symptoms, and psychological state based on clinical practice, expert interviews, and previous research. The basic information of the participating experts is shown in Table 1.

**Table 1:** The basic information of experts with scale consultation

Item		Number (%)
Gender	Male	16（72.7）
	Female	6（27.3）
Highest Degree	Doctoral	17（77.3）
	Master	1（4.5）
	Bachelor	4（18.2）
Professional Titles	Senior Professional	10（45.0）
	Deputy Senior	8（36.8）
	Intermediate professional	4（18.2）
Years of Work Experience	>20 years	15（68.2）
	≤20 years	7（31.8）
Specialty	Thoracic Surgery	13（59.1）
	Psychology	3（13.7）
	Nutrition	1（4.5）
	Rehabilitation Medicine	1（4.5）
	Nursing	4（18.2）

Expert Correspondence and Scale Formulation

There were two rounds of expert correspondence in total. The first round of expert correspondence included 22 experts, and the second round of expert correspondence included 19 experts. The content of the correspondence inquiry included expert information, authority evaluation form, item relevance evaluation, importance evaluation, and modification opinions. The authority evaluation form included the expert's basis for judgment and familiarity with each item. The relevance of the item referred to the degree of relevance of the item to the research content. A score of 1 represented "irrelevant", a score of 2 represented "weak relevance", a score of 3 represented "general relevance", a score of 4 represented "strong relevance", and a score of 5 represented "very relevant". In the importance assessment, 1 represented "very unimportant", a score of 2, 3, and 4 represented increasing importance, and 5 represented "very important". Expert revision opinions could suggest deleting, adding, or adjusting rhetoric.

Research Indicators

Active coefficient: The response rate of the expert consultation questionnaire indicates the level of cooperation and engagement from the experts. Evaluation criteria: A response rate of >70% indicates a high level of motivation from the experts, a response rate between 60% and 70% indicates a moderate level of motivation, and a response rate of at least >50% indicates an acceptable level of participation.

Authority coefficient: The authority coefficient (Cr) is an indicator used to measure the authority of experts. It is composed of two factors: the basis for the expert's judgment on the consulting issue (Cs) and the degree of familiarity (Ca). The calculation formula is Cr = (Cs + Ca) / 2 [10]. The basis for judgment is calculated from four dimensions: theoretical analysis, practical experience, peer understanding, and personal intuition. The degree of influence of each dimension on judgment is divided into large, medium, and small. Based on the degree of influence, the value assignment for each judgment dimension is as follows: theoretical analysis (0.3, 0.2, 0.1), practical experience (0.5, 0.4, 0.3), peer understanding (0.1, 0.1, 0.1), personal intuition (0.1, 0.1, 0.1). The degree of familiarity is divided into five levels: very familiar, relatively familiar, generally familiar, not very familiar, and unfamiliar, with values assigned as 1.0, 0.8, 0.6, 0.4, and 0.2, respectively. If the authority coefficient is ≥0.7, the authority of the expert is considered acceptable [11,12].

Coefficient of Kendall (KW): The coordination coefficient represents the consistency of all experts' evaluations of all indicators and is measured using Kendall's concordance coefficient (W). The value of W ranges between 0 and 1, with a higher value indicating a better degree of coordination. After conducting the coordination coefficient test, if the p-value is less than 0.05, the result is considered desirable, indicating that the experts' evaluations of all indicators are consistent [13].

Coefficient of variation (CV): The coefficient of variation represents the consistency of experts' evaluation of a single indicator. It is calculated as the ratio of the mean importance score of an indicator to the standard deviation. A smaller value indicates a higher consistency of expert opinions and a smaller degree of dispersion [14].

Importance score: The importance score represents the arithmetic mean of all experts' ratings regarding the importance of an indicator. A higher value indicates a greater level of importance for the indicator within the indicator system.

Scale Reliability and Validity Test

Research subjects: A total of 200 patients who underwent lung cancer surgery were included in the study. The patients were selected from three medical groups at the Lung Cancer Center of West China Hospital, Sichuan University, between August and November 2022. Patients should meet all the inclusion criteria, those inclusion criteria were as follows: 1. Confirmed diagnosis of lung cancer through postoperative pathology; 2. Willingness to participate in the study; 3. Age between 18 and 80 years old and underwent thoracoscopic lung surgery; 4. Patients who regained consciousness after surgery and were able to complete the questionnaire survey.

The exclusion criteria were as follows: 1. Presence of other serious medical conditions; 2. Patients with mental illness or impaired consciousness; 3. Patients under the protection of family members; 4. Intraoperative blood loss exceeding 1000 ml, conversion to thoracotomy, or severe postoperative complications requiring additional medical interventions (such as re-insertion of chest drainage tube or surgery); 5. Refusal to participate in the study; 6. Failure to complete the questionnaire. Meeting one of these criteria should exclude them from this research.

Questionnaire entry selection:* *The questionnaire items were screened using the critical ratio method and correlation coefficient method. The procedure was as follows: 1. Critical ratio method: The total scores of all participants on the pre-test scale were sorted from high to low. Those scoring in the top 27% were categorized as the high group while those scoring in the bottom 27% were categorized as the low group. The scores of the high and low groups for each item were statistically analyzed using a t-test. 2. Correlation coefficient method: Correlation analysis was conducted between the score of each symptom item and the total score.

Evaluation of the reliability and validity of the scale: Reliability evaluation involves using Cronbach's alpha coefficient to assess the internal consistency reliability among each symptom item. Content validity is assessed through the average scale level content validity index (S-CVI/Ave). Construct validity is evaluated using exploratory factor analysis. The applicability of the factor analysis method is tested using the KMO (Kaiser-Meyer-Olkin) value and Bartlett's sphericity test. Subsequently, principal component analysis and the varimax orthogonal rotation method are employed [14].

Statistical Methods

This study utilized SPSS 25.0 software (IBM Corp., Armonk, NY, USA) for data processing and statistical analysis. Item analysis was conducted using independent sample t-tests and two-sample correlation tests (Pearson correlation coefficient). Internal consistency reliability was assessed using Cronbach's α coefficient analysis. Content validity was evaluated using CVI while construct validity was evaluated through principal component analysis with maximum variance rotation factor analysis. Statistical significance was determined when p<0.05.

Ethical Review

Institutional review board approval was given by the Biomedical Research Ethics Committee of West China Hospital of Sichuan University (NO. 2019-1115).

## Results

Analysis of the results of correspondence consultation with experts on scale formulation

Experts’ Enthusiasm

In the first round, a total of 22 questionnaires were distributed, and all 22 were recovered. All of the returned questionnaires were complete and valid, resulting in a recovery rate of 100%. Additionally, all 22 experts provided revision opinions. In the second round, 19 questionnaires were distributed, and all 19 were recovered. Similar to the first round, all of the returned questionnaires were complete and valid, resulting in a recovery rate of 100%.

Expert Authority

The overall authority coefficient of the first and second rounds of expert correspondence was 0.87 and 0.88, respectively, indicating a high level of authority. Furthermore, the authority coefficient of each questionnaire item was >0.7. The detailed results are presented in Table 2.

**Table 2:** Authority coefficient and coefficient of variation of scale expert questionnaire survey

NO.	Item	Authority coefficient（Cr）			Coefficient of variation（CV）	
		First round	Second round		First round	Second round
1	Eating	0.88		0.93	0.16	0.06
2	Dressing	0.79		0.93	0.28	0.00
3	Early Ambulation	0.92		0.91	0.09	0.11
4	Defecation	0.83		0.95	0.11	0.06
5	Cough	0.95		0.91	0.07	0.09
6	Pain	0.95		0.85	0	0.11
7	Sleep	0.91		0.85	0.09	0.11
8	Fatigue	0.82		0.82	0.18	0.14
9	Dizziness	0.78		0.82	0.28	0.15
10	Negative Emotion	0.85		0.79	0.15	0.21

Consistency of Expert Opinions

After conducting the Kendall coordination coefficient W test, the coordination coefficients of the two rounds of expert correspondence were 0.40 (*P*<0.001) and 0.48 (P<0.001), respectively. The coefficients of variation for each item were all <0.30, indicating a high level of consistency in the overall opinions of the two rounds of expert consultation. The results are presented in Table 2.

General information of survey respondents

A total of 200 patients participated in this study, and 190 valid questionnaires were collected, resulting in an effective response rate of 95%. Among the participants, 65 were male and 125 were female. The age range was 21-80 years old, with a mean age of (50.54±13.19) years.

Item screening analysis results

The critical ratio method was employed to examine the score difference of each item between the high and low groups. Statistically significant differences were observed in all items (P<0.05).

The correlation between each item and the total score (Pearson correlation coefficient) was calculated, revealing a significant correlation. Please refer to Table 3 for more details.

**Table 3:** Correlation between questionnaire items and expert survey results

NO.	Item	Expert correspondence score（Mean±SD）			Content validity	Pearson correlation coefficient	*P* values
		First round	Second round		I-CVI		
1	Eating	4.57±0.71		4.78±0.42	1.00	0.85	＜0.05
2	Dressing	3.65±1.00		3.78±0.79	0.89	0.70	＜0.05
3	Early Ambulation	4.78±0.41		4.89±0.31	1.00	0.84	＜0.05
4	Defecation	4.39±0.49		4.33±0.47	1.00	0.74	＜0.05
5	Cough	4.87±0.34		4.84±0.45	1.00	0.89	＜0.05
6	Pain	5.00±0.00		4.89±0.31	1.00	0.84	＜0.05
7	Sleep	4.78±0.41		4.44±0.50	1.00	0.48	＜0.05
8	Fatigue	4.13±0.74		4.11±0.57	0.89	0.62	＜0.05
9	Dizziness	3.70±1.04		4.22±0.63	0.89	0.58	＜0.05
10	Negative Emotion	4.39±0.64		4.56±0.50	1.00	0.57	＜0.05

Scale reliability and validity test

Internal Consistency Reliability

The Cronbach's α coefficient of the scale is 0.801.

Content Validity

 Based on the results of expert correspondence, the scale's content validity index (S-CVI) was calculated to be 0.97. The content validity index (I-CVI) of each item is presented in Table 3.

Construct Validity

Exploratory factor analysis was conducted to assess the suitability of the data for factor analysis using the Kaiser-Meyer-Olkin (KMO) test and Bartlett's sphericity test (KMO=0.775, χ2=439.00, P<0.001). The results indicated that the data were suitable for factor analysis. Principal component analysis with varimax orthogonal rotation was performed, resulting in the extraction of two common factors. The eigenvalues of the common factors 1 and 2 were 3.257 and 1.352, respectively, both exceeding 1. The cumulative variance contribution rates for these factors were 32.57% and 13.52%, respectively. The factor loadings for each item are presented in Table 4.

**Table 4:** Results of the factor load and ANOVA for each item after surgery ANOVA: analysis of variance

NO.	Item	Communality		Factor loading			
				Common factor 1		Common factor 2	
1	Eating		0.479		0.679		-0.135
2	Dressing		0.631		0.793		0.040
3	Early Ambulation		0.660		0.808		0.086
4	Defecation		0.657		0.808		0.063
5	Cough		0.765		0.013		0.604
6	Pain		0.468		0.367		0.577
7	Sleep		0.563		-0.199		0.724
8	Fatigue		0.462		0.571		0.158
9	Dizziness		0.614		0.582		-0.260
10	Negative Emotion		0.409		0.634		-0.088

## Discussion

The rapid development of imaging and minimally invasive surgery in China has allowed lung cancer patients to receive an earlier diagnosis and undergo less invasive procedures, leading to extended patient survival and reduced hospitalization costs and length of stay [15-17]. However, as people have raised their expectations for the comfort of hospitalization, the primary challenge facing thoracic surgeons has shifted from simply prolonging patients' lives to ensuring a better quality of life for patients while also extending their lifespan [17-19]. In light of these advancements, medical professionals and researchers in China are actively exploring the field of accelerated rehabilitation and have achieved significant progress [20,21]. How to enhance patient comfort during hospitalization is a specific area of focus within the concept of ERAS. To effectively improve patient comfort, it is necessary to have assessment tools that are suitable for China's national conditions and cultural background.

The most commonly used comfort assessment scale in China is the GCQ comfort status scale. It has been widely adopted for assessing the comfort of inpatients in various departments [22]. The scale has undergone further revisions to ensure its suitability for China's national conditions and cultural background [23]. When we attempted to use the GCQ scale to assess patient comfort, we encountered a low completion rate due to the large number of questionnaire items. Despite our efforts to closely translate the GCQ scale into Chinese, patients often expressed confusion about the questions during actual usage, likely due to cultural differences. As a result, we endeavored to reformulate the postoperative comfort assessment scale specifically for lung cancer patients.

During the scale development process, we conducted two rounds of expert correspondence. All experts completed the questionnaire and provided valuable feedback. After evaluating the experts' authority and the consistency of their opinions, it was found that the participating experts in both rounds had a high level of authority and opinion consistency. The scale's content validity index (S-CVI), calculated based on the results of expert correspondence, was 0.97, indicating good content validity. Furthermore, each item showed a strong correlation with the total score. During the reliability and validity testing process, the test-retest reliability was not analyzed due to the constantly changing condition and treatment of the patients, as well as the short duration of the postoperative hospital stay. Generally, a Cronbach's α value greater than 0.80 indicates excellent internal consistency. In this case, the Cronbach's alpha coefficient of the scale is 0.801, indicating good internal consistency reliability [24]. There are three criteria for assessing the structural validity of a questionnaire [25,26]. The first one is the common factors should align with the component areas of the structural hypothesis at the time of questionnaire design, and the cumulative variance contribution rate of the common factors should be at least 40%. Second, each item should have higher loading values (greater than 0.4) on its corresponding common factor while the loading values for other common factors should be lower. If an item has low loading values on all factors, it indicates that its intended meaning is unclear and should be revised or removed. The last one is that the variance explained by the common factors should be greater than 0.4, meaning that more than 40% of the variance in each item can be attributed to the common factors.

The limitations of this study and planned improvements in the next step are as follows. First, the patients were not assessed with the GCQ scale simultaneously. Given that the GCQ scale is currently a widely used comfort assessment tool, evaluating the same patient using two scales simultaneously would be helpful in assessing criterion validity. Second, this study aimed to assess three dimensions: self-care ability, postoperative symptoms, and mental state. However, the limited number of questions designed for the mental state dimension affected the structural validity and internal consistency reliability of the questionnaire. In summary, the postoperative comfort scale can effectively measure the comfort level of lung cancer patients during their postoperative hospitalization, serving as a valuable clinical tool for comfort assessment. However, further revisions are needed to enhance the content of the scale items.

## Conclusions

Based on the commonly used GCQ scale, and the unique and common symptoms experienced by patients after lung cancer surgery, we condensed 30 items from the physiological, psychological, sociocultural, and environmental dimensions. Using the Delphi method, we selected 10 items to form the postoperative comfort scale specifically designed for lung cancer patients. This scale has demonstrated high reliability and validity. Furthermore, our study identified pain and mobility (early ambulation or getting out of bed) as the primary factors influencing the postoperative comfort of lung cancer patients.
